# Does testosterone mediate the relationship between vitamin D and prostate cancer progression? A systematic review and meta-analysis

**DOI:** 10.1007/s10552-022-01591-w

**Published:** 2022-06-26

**Authors:** Luke A. Robles, Sean Harrison, Vanessa Y. Tan, Rhona Beynon, Alexandra McAleenan, Julian PT. Higgins, Richard M. Martin, Sarah J. Lewis

**Affiliations:** 1 Department of Population Health Sciences, Bristol Medical School, University of Bristol, Bristol, England; 2 Medical Research Council Integrative Epidemiology Unit, University of Bristol, Bristol, England; 3 NIHR Bristol Biomedical Research Centre, University Hospitals Bristol and Weston NHS Foundation Trust and University of Bristol, Bristol, England

**Keywords:** Vitamin D, Testosterone, Prostate cancer, Progression, Meta-analysis

## Abstract

**Purpose:**

Observational studies and randomized controlled trials (RCTs) have shown an association between vitamin D levels and prostate cancer progression. However, evidence of direct causality is sparse and studies have not examined biological mechanisms, which can provide information on plausibility and strengthen the evidence for causality.

**Methods:**

We used the World Cancer Research Fund International/University of Bristol two-stage framework for mechanistic systematic reviews. In stage one, both text mining of published literature and expert opinion identified testosterone as a plausible biological mechanism. In stage two, we performed a systematic review and meta-analysis to assess the evidence from both human and animal studies examining the effect of vitamin D on testosterone, and testosterone on advanced prostate cancer (diagnostic Gleason score of ≥ 8, development of metastasis) or prostate cancer-specific mortality.

**Results:**

A meta-analysis of ten human RCTs showed evidence of an effect of vitamin D on total testosterone (standardised mean difference (SMD) = 0.133, 95% CI =  − 0.003–0.269, I^2^ = 0.0%, *p* = 0.056). Five human RCTs showed evidence of an effect of vitamin D on free testosterone (SMD = 0.173, 95% CI =  − 0.104–0.450, I^2^ = 52.4%, *p* = 0.220). Three human cohort studies of testosterone on advanced prostate cancer or prostate cancer-specific mortality provided inconsistent results. In one study, higher levels of calculated free testosterone were positively associated with advanced prostate cancer or prostate cancer-specific mortality. In contrast, higher levels of dihydrotestosterone were associated with lowering prostate cancer-specific mortality in another study. No animal studies met the study eligibility criteria.

**Conclusion:**

There is some evidence that vitamin D increases levels of total and free testosterone, although the effect of testosterone levels within the normal range on prostate cancer progression is unclear. The role of testosterone as a mechanism between vitamin D and prostate cancer progression remains inconclusive.

**Supplementary Information:**

The online version contains supplementary material available at 10.1007/s10552-022-01591-w.

## Introduction

Prostate cancer is the most common cancer in men in the UK with approximately 48,500 newly diagnosed cases and is the second cause of male-related cancer mortality in the UK [1]. Prostate-specific antigen (PSA) screening has led to many men being diagnosed with localised prostate cancer [2]. However, although many men are diagnosed with localised prostate cancer in old age, the majority of these tumours do not progress to become advanced tumours and the use of PSA screening to identify men with localised prostate cancer has been shown to have little effect on prostate cancer-specific mortality when compared to usual care (Incidence rate ratio = 0.96, 0.85–1.08) [3]. In a large randomized controlled trial of men diagnosed with localised prostate cancer who were followed up over a median of 10 years, only around 8% of men were found to have evidence of disease progression over this time period [4]. With many men diagnosed and living with prostate cancer, it is important to identify modifiable exposures that increase men’s risk of prostate cancer progression. The association of vitamin D with cancer progression outcomes, including prostate cancer, has received much attention [5–7].

Vitamin D, a fat-soluble vitamin, is essential to the absorption of calcium from the gut into the bloodstream and regulates circulating phosphate levels and bone mineralisation. Vitamin D is available through three sources: sunlight, plant-based and fortified foods (e.g., breakfast cereals), and supplements, and is hydroxylated in the liver and kidneys to produce calcitriol (the active hormone) [8].

Evidence exists of an association between vitamin D on prostate cancer progression and mortality. For example, a meta-analysis of cohort studies with 7808 participants found higher circulating 25(OH)D vitamin D levels to be associated with reduced risk of prostate cancer-specific mortality (Hazard ratio = 0.91, 95% CI = 0.87–0.97, *p* = 0.002, I^2^ = 53.4%) [9]. Intervention studies have found that vitamin D supplementation results in a lower number of repeat positive biopsy cores (55% reduction [10]) at a one year follow-up and lower prostate-specific antigen levels [11] at 6–8 weeks follow-up among men with low and intermediate stage prostate cancer.

The strength of evidence for, and plausibility of, an effect of vitamin D on prostate cancer progression (i.e., Gleason scores of ≥ 8, metastasis, prostate cancer-specific mortality) may be improved if studies which examine potential underpinning mechanistic pathways are considered. In the current study, we used the World Cancer Research Fund International/University of Bristol two-stage mechanistic review framework to synthesise evidence from a wide range of different study types, including human and animal studies [12]. Stage one involved identifying a relevant biological mechanism for the vitamin D—prostate cancer progression association using text mining approaches. For stage two, the evidence for the mechanism in relation to both the vitamin D exposure and the prostate cancer progression outcome was systematically reviewed. Further details of the methodology are published elsewhere [12].

Testosterone, a male sex hormone produced by the testis and adrenal glands with a critical role in driving cell division in the prostate gland, was chosen as a potential mechanism from our stage one exploration for two main reasons. First, text mining analyses showed that there was a greater quantity of evidence linking testosterone with both vitamin D and prostate cancer than for other potential mechanisms. Second, there is biological plausibility for testosterone having a causal role in prostate cancer; studies by Huggins and colleagues [13, 14] found that testosterone administered after surgical castration of men with metastatic prostate cancer resulted in increased rates of prostate cancer progression. Androgen deprivation therapy (ADT) is subsequently used clinically to reduce testosterone production in the treatment of prostate cancer. Further information on how we selected testosterone as a mechanism in stage 1 of this review is provided in reference 15.

Stage two of our systematic review of mechanisms aimed to synthesize human and animal studies to investigate whether there is evidence that an association of vitamin D on prostate cancer progression could be via an effect of vitamin D on circulating levels of testosterone within the normal range.

## Methods

The protocol of this systematic review can be found elsewhere [15]. We conducted the systematic review using the World Cancer Research Fund International/University of Bristol two-stage mechanistic review framework [12]. We reported this systematic review in accordance with the Preferred Reporting Items for Systematic review and Meta-Analysis guidelines [16]. A populated checklist for this review has been provided in Supplementary file 1.

### Participants

For the studies linking vitamin D to testosterone, we included those on men only or studies presenting data stratified by sex. For the studies linking testosterone to prostate cancer progression outcomes, we included men with pre-diagnostic testosterone concentrations or men diagnosed with localised prostate cancer and a measurement of testosterone at baseline.

### Exposures

#### Vitamin D

We included any duration, frequency, and dose of vitamin D, including nutrition supplements, for intervention studies examining the vitamin D-testosterone association. There were no restrictions on vitamin D exposures in observational studies.

#### Testosterone

Eligible human studies for the testosterone-prostate cancer analyses included those which measured total testosterone, free testosterone, bioavailable testosterone, or dihydrotestosterone. Most circulating testosterone is bound to two proteins in the blood—albumin and sex hormone binding globulin (SHBG)—and is measured directly in a blood sample as total testosterone. Free testosterone is a fraction of circulating testosterone (approximately 2%) that is unbound to these two proteins and is measured either directly from a blood sample or can be calculated using values of albumin and SHBG. Bioavailable testosterone is the sum of free testosterone and albumin-bound testosterone. Approximately 10% of testosterone is converted to a hormone dihydrotestosterone by certain tissues of the body, including the prostate gland, and is responsible for the growth of the prostate.

Animal studies which examined endogenous testosterone levels on prostate cancer progression association were eligible.

### Outcomes

Outcomes of interest were: (i) total tostestorone, free testosterone, and dihydrotestosterone concentrations for vitamin D-testosterone association studies; and (ii) a diagnostic Gleason score of ≥ 8, development of metastasis, and prostate cancer-specific mortality, for studies of testosterone-prostate cancer progression.

### Eligible studies

We included original studies published in peer-reviewed articles. There was no restriction on the publication date of the articles or language. Eligible studies included observational studies (prospective cohorts, nested case–control studies), Mendelian randomization studies, human experimental studies (randomised controlled trials, cross-over studies), and animal studies. To evaluate the testosterone-prostate cancer progression association, we limited observational studies to those with a follow-up of at least 2 years or with a median or mean of 5 years between the measurement of testosterone and a diagnosis of advanced cancer or prostate cancer-specific mortality. As we were interested in the effect of normal variation in endogenous testosterone levels on measures of prostate cancer progression and to avoid the possibility of reverse causation, we excluded studies that examined testosterone treatment effects on prostate cancer progression, in particular the effects of ADT. Both vitamin D and testosterone concentrations vary by age. Therefore, observational studies that did not adjust for age in their analyses or where a large difference in age were observed were excluded from the review.

We excluded cross-sectional and retrospective case-only study designs to avoid reverse causation. We also excluded in vitro and xenograft studies, and animal studies presenting cell line data only, as these designs provide weak evidence on mechanisms operating in humans.

### Literature searches

We searched the following electronic bibliographic databases for relevant published articles without year or language restrictions: PubMed (from inception to May 2020); Ovid MEDLINE (1946 to May 2020); Ovid EMBASE (1980 to May 2020); and BIOSIS Citation Index (1969 to May 2020). Two sets of searches were performed: (1) studies that linked circulating vitamin D to circulating testosterone; and (2) studies that linked circulating testosterone to measures of prostate cancer progression (i.e., Gleason score of ≥ 8, development of metastases, prostate cancer-specific mortality). Search strategies included standard controlled vocabulary (MeSH and Emtree), text words, and keywords, and were amended to accommodate the individual requirements of each bibliographic database. An information specialist with experience of conducting systematic reviews was consulted to advise on the search strategies for each database, which are shown in Supplementary file 2.

We searched the reference lists of each included article, relevant systematic review articles, and commentaries and letters found within the electronic searches.

### Study selection

All titles and abstracts yielded from each search were initially screened for duplicates based on titles, author names, page numbers, years of publication, and journal names. All titles and abstracts were then screened against the inclusion criteria independently by two of four authors (LAR, VYT, RB, SJL). If an abstract was not available or provided insufficient information to inform a screening decision, the full text article was retrieved. The full text of potentially eligible articles identified from the title and abstract screening was retrieved and assessed against the eligibility criteria. Full text articles were screened by two authors (LAR, SH, SJL) and included if a consensus decision was reached. Disagreements in full text screening were resolved through discussion.

### Data extraction

Data on the following characteristics were extracted from each included study: study location, demographics (age, ethnicity), study design, exposure measurement (including type, dose, and duration for vitamin D; serum concentration for total and free testosterone and dihydrotestosterone), length of follow-up, and measures of prostate cancer progression (i.e., Gleason score, metastases, and prostate cancer-specific mortality). Statistical data were extracted including: sample size, effect estimate (mean, standard deviation, median, interquartile range, *p* value, odds ratio, 95% confidence intervals), and whether studies adjusted for age in their analysis. Data were extracted by one author (LR) and checked for accuracy by another author (SH). Discrepancies were resolved through discussion among the authors.

### Risk of bias in individual studies

We performed risk of bias (RoB) assessments on each included study. We used the Risk of Bias 2 (RoB 2) tool [17] to assess human randomised controlled trials. The RoB 2 tool assessed the overall RoB for each study using the following rating: high risk of bias, some concerns, low risk of bias, or no information. For human cohort studies, we used a tool developed for a previous systematic review of mechanistic studies [18] that included domains of assessment from the ROBINS-I tool [19] and questions from the CASP cohort assessment [20]. Each tool evaluated bias due to: confounding, selection of participants, missing data, outcome and exposure measurement, and selective reporting of results. All human cohort studies were considered initially to be at moderate RoB before the assessments were performed and remained at moderate risk unless subsequently found to be at a higher RoB. This is because confounding cannot be fully controlled for within these study designs. We did not perform a risk of bias assessment on Mendelian randomisation studies as there is no risk assessment tool available for these studies at present.

### Assessment of reporting bias

We assessed the potential for publication bias using a funnel plot and Egger’s test for the vitamin D- testosterone studies where we had more than 10 studies [21].

### Grade assessments

We assessed the certainty of evidence for each association using the Grading of Recommendations Assessment, Development and Evaluation system (GRADE; [22]). RCTs were given an a-priori ranking of high and observational studies a ranking of low certainty of evidence. Rankings were subsequently downgraded based on the following five categories: (1) risk of bias; (2) inconsistency of results; (3) indirectness of evidence; (4) imprecision; and (5) reporting bias. A final certainty of evidence rating, which ranged from high, moderate, low, and very low rating, was given to each study after a consensus was reached among four authors (JPTH, RMM, SJL, LAR).

### Statistical analyses

We performed a meta-analysis of sufficiently similar studies using STATA version 15 [23]. We estimated the standardised mean difference (SMD) and standard error for each vitamin D-testosterone association study. We calculated the SMD by calculating the difference in means (MD) (intervention mean minus control mean) at final follow-up and dividing by the average standard deviation (SD) of the exposure (SD_e_) and control (SD_c_) groups (i.e., average SD = (SD_e_^2^ + SD_c_^2^/2)^2^. We estimated standard deviations where studies presented interquartile ranges [24]. All vitamin D and testosterone concentrations included in the meta-analyses were converted to nanograms per millilitre (ng/ml) if they were not reported as such. We performed random effects and fixed effects meta-analyses using the metan STATA command [25]. The degree of inconsistency across studies was assessed using the I^2^ statistic [26].

## Results

**Fig. 1:**
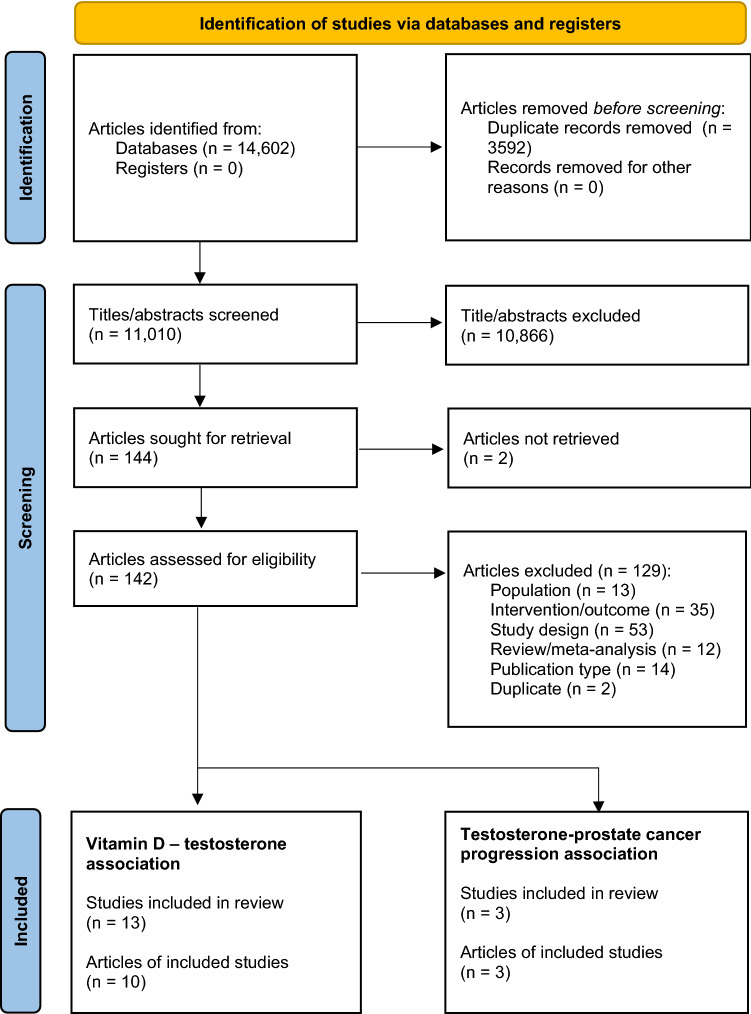
PRISMA flow diagram of database searches

Electronic searches of all four databases identified 14,602 articles. Duplicates (*n* = 3592) were removed, leaving 11,010 articles for screening of titles and abstracts. Full text articles were retrieved (*n* = 142) and assessed for eligibility, which resulted in the identification of 16 studies for data extraction and RoB assessments. Hand searching of included study reference lists did not find any additional articles. Figure 1 presents a PRISMA flowchart showing the route to identification of the selected studies via the database searches.

### Vitamin D-testosterone studies

**Fig. 2:**
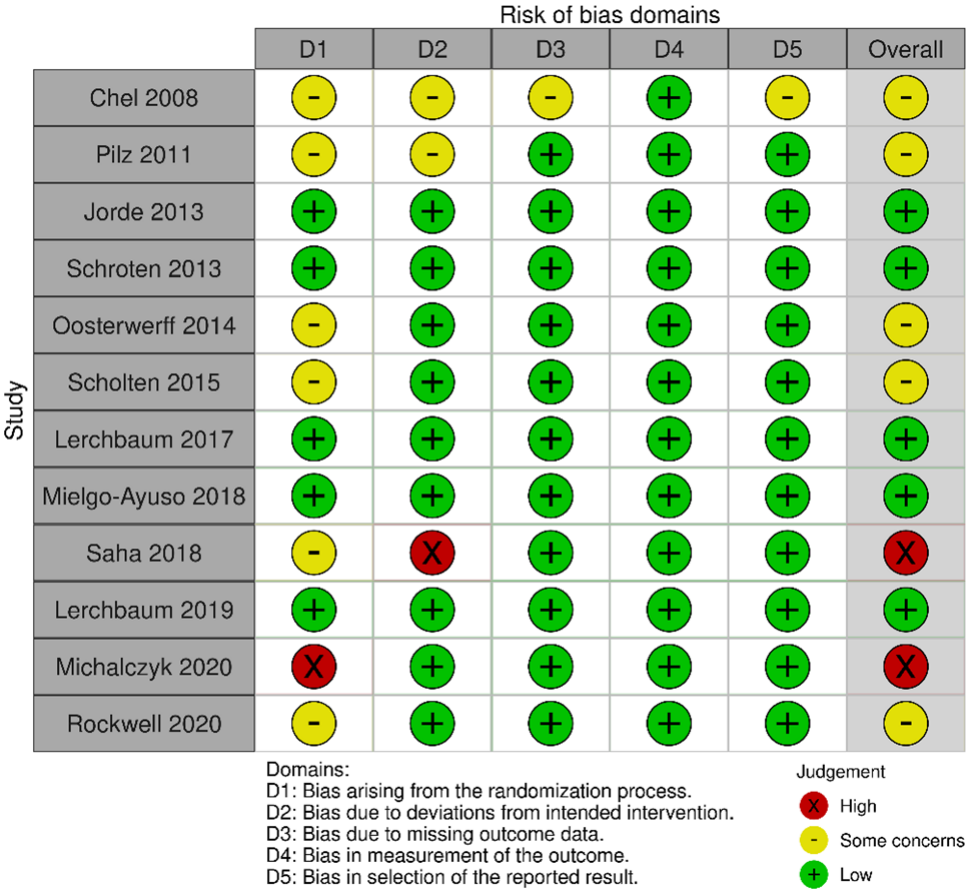
Risk of bias of vitamin D-testosterone studies

Thirteen studies examined the vitamin D-testosterone association. Twelve of these studies were human randomised controlled trials (3 factorial; 9 parallel group) that examined the effects of vitamin D supplementation compared to placebo. The other study was a Mendelian randomisation study [27]. All 13 measured total testosterone (including 1 using a genetic risk score as a proxy measure), five measured free testosterone, and one measured bioavailable testosterone. Two out of the 12 RCTs were judged at high risk of bias. One of these performed a per-protocol analysis only [28] and the other reported higher levels of vitamin D (a greater difference than would be expected by chance) in the intervention group compared to the control groups at baseline [29]. Five studies were judged as having some concerns of bias arising from the randomisation process and deviations from the intended interventions. The remaining five studies had a low risk of bias (Fig. 2). There were no animal studies identified which examined the link between vitamin D and endogenous testosterone concentrations within normal ranges. From the above studies, we obtained ten effect sizes for total testosterone [29–35], 5 for free testosterone [29, 30, 32–34], and one for bioavailable testosterone [30]. Table 1 presents the descriptive characteristics of the vitamin D-testosterone studies.

**Table 1:** Characteristics of included human vitamin D-testosterone association studies

Study	Author (year) location	Study design	Total sample size	Participant characteristics	Age (years)	Vitamin D (dose and frequency) or exposure measurement	Final follow-up (weeks)	Outcome of interest
1	Chel (2008) The Netherlands	RCT	43	Men in nursing home	71–97 (range	18,000 IU/month	16	TT
2	Pilz (2011) Germany	RCT	54	Men from weight loss program	20–49 (range)	3,332 IU daily	52	TT, FT, BT
3	Jorde (2013)	RCT (pooled analysis)	282	Men recruited from 3 intervention studies: an obesity, insulin sensitivity, and depression intervention study	51 (mean)	20 000 IU/week and 40,000 IU/week (Obesity study vitamin D interventions combined) 40,000 IU (Insulin study) 40,000 IU (Depression study	52 weeks (Obesity study) 24 (Insulin study) 24 (Depression study	TT, FT
4	Schroten (2013) The Netherlands	2 × 2 factorial RCT	86	Men with chronic heart failure	42–86	2000 IU daily	6	TT
5	Oosterwerff (2014) Amsterdam	RCT	32	Men overweight with prediabetes	20–70	1,200 IU daily	16	TT
6	Scholten (2015)^a^ USA	2 × 2 factorial RCT	23	Physically active men	25–42 (range)	4,000 IU daily	8	TT
7	Lerchbaum (2017) Austria	RCT	98	Healthy men with normal TT levels	39 (mean)	20,000 IU/week	12	TT, FT
8	Mielgo-Ayuso (2018) Spain	RCT	36	Elite male rowers	27 (mean)	3,000 IU daily	8	TT
9	Saha (2018) India	2 × 2 factorial RCT	92	Graduate and postgraduate students	20 (mean)	60,000 IU/week for 8 week, 60,000 IU/fortnightly for 4-months (with placebo calcium tablets)	24	TT
10	Lerchbaum (2019) Austria	RCT	94	Men with low TT levels	49 (median)	20,000 IU/week	12	TT, FT
11	Michalczyk (2020) Poland	RCT	28	Elite football players	Not reported	6,000 IU daily	6	TT, FT
12	Rockwell (2020)^a^ USA	RCT	6	College swimmers	Not reported	5,000 IU daily	12	TT, FT
13	Chen (2019)^a^ China	Mendelian Randomization	4,254	Healthy men	56 (mean)	Vitamin D genetic risk score (4 SNP: rs12785878, rs10741657, rs2282679, rs6013897)	NA	TT

^a^ = Study not included in meta-analysis

**Fig. 3:**
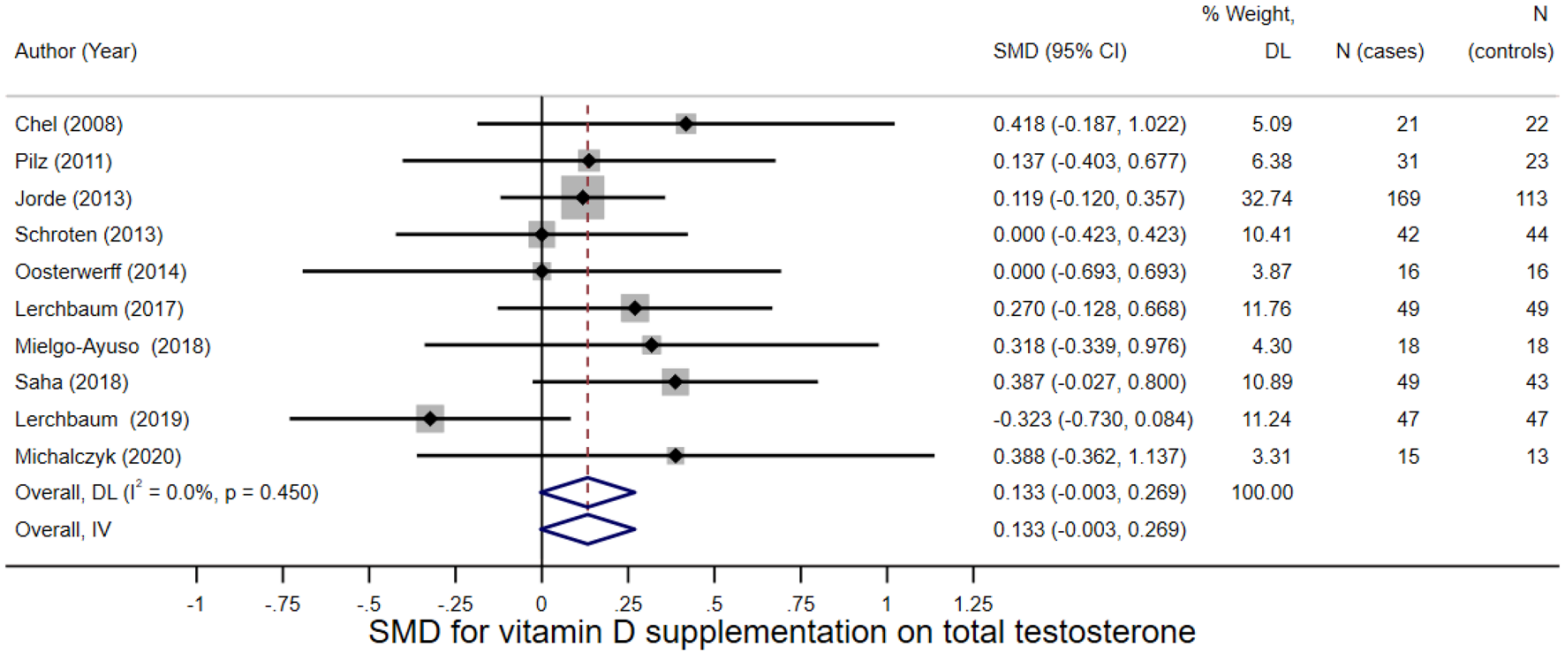
Forest plot of vitamin D—total testosterone studies

A meta-analysis of the 10 individual study effect sizes for total testosterone found evidence of an effect of vitamin D on total testosterone. Each 1 SD increase in vitamin D was associated with an increased level of total testosterone by 0.133 of an SMD (95% CI =  − 0.003–0.269, I^2^ = 0.0%, *p* = 0.056) (Fig. 3). There was no evidence of small study effects indicating publication bias (Egger’s test *p* = 0.535). Table 2 present the effect sizes and standard errors of the vitamin D-testosterone studies.

**Table 2:** Summary of results reported in the human vitamin D-testosterone studies included in the meta-analyses

Author	Year	Outcome (ng/ml)	N total sample	Intervention			Control			p value1	Standardised mean difference	SE
				N cases	Mean/median	SD	N controls	Mean/median	SD			
Chel	2008	TT	43	22	0.14	0.92^a^	21	− 0.29	1.14^a^	NR	0.42	0.31
Pilz 1	2011	TT	54	31	3.86	1.36	23	3.66	1.59	NR	0.14	0.28
Pilz 2	2011	FT	54	31	0.08	0.03	23	0.08	0.03	NR	− 0.12	0.28
Pilz 3	2011	BT	54	31	1.80	0.58	23	1.90	0.67	NR	− 0.16	0.28
Jorde 1	2013	TT	282	169	− 0.03	1.07	113	− 0.14	0.87	NR	0.12	0.12
Jorde 2	2013	FT	282	169	0.00	0.03	113	0.00	0.02	NR	0.13	0.12
Schroten	2013	TT	86	44	0.00	0.89^a^	42	0.00	0.88^a^	NR	0.00	0.22
Oosterwerff	2014	TT	32	16	0.00	0.47^a^	16	0.00	0.94^a^	NR	0.00	0.35
Lerchbaum 1	2017	TT	98	49	5.60	0.88^a^	49	5.28	1.41^a^	0.50	0.27	0.20
Lerchbaum 2	2017	FT	97	48	0.10	0.09^a^	49	0.10	0.03^a^	0.69	0.10	0.20
Mielgo-Ayuso	2018	TT	36	18	4.73	1.28	18	4.37	0.96	0.85‡	0.32	0.34
Saha	2018	TT	92	49	5.91	1.73	43	5.31	1.38	0.42	0.39	0.21
Lerchbaum 1	2019	TT	94	47	3.69	1.17^a^	47	4.10	1.32^a^	0.78	− 0.32	0.21
Lerchbaum 2	2019	FT	92	46	0.08	0.03^a^	46	0.08	0.03^a^	0.83	0.07	0.21
Michalczyk	2020	TT	28	15	8.15	0.92	13	7.67	1.47	< 0.01	0.39	0.38
Michalczyk	2020	FT	28	15	0.03	< 0.01	13	0.02	0.01	< 0.01	1.27	0.41

^a^ = SD estimated using interquartile range

**Fig. 4:**
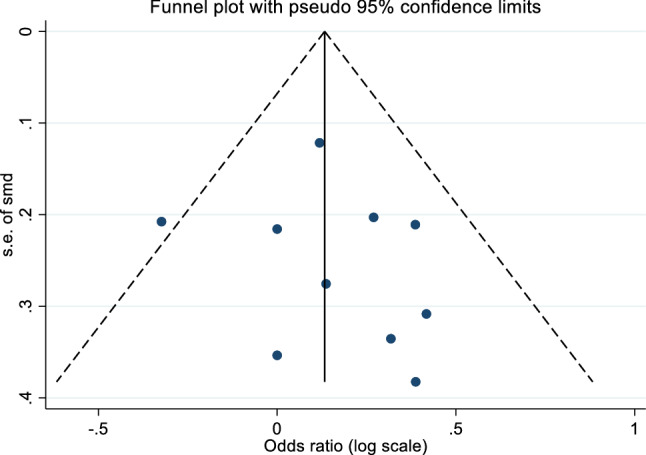
Funnel plot of vitamin D—total testosterone studies

**Fig. 5:**
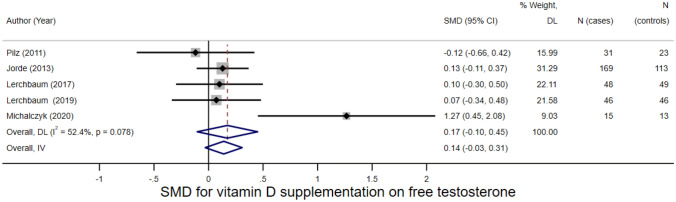
Forest plot of vitamin D—free testosterone studies

Whilst the evidence for free testosterone was not strong, the effect was in the same direction and of a similar magnitude. In a meta-analysis of five studies which had assessed this, the increase in the SMD for free testosterone was 0.173 (95% CI =  − 0.104–0.450, I^2^ = 52.4%, *p* = 0.220) (Fig. 4). There was no evidence of small study effects indicating publication bias (Egger’s test *p* = 0.405 (Fig. 5).

One RCT [30] found little evidence of an effect of vitamin D supplementation on bioavailable testosterone (SMD =  − 0.156, 95% CI =  − 0.696–0.384), but the effect estimate for this was in the opposite direction to those for the meta-analysed studies of total testosterone and free testosterone.

We were not able to extract effect sizes related to the effect of vitamin D from two studies of total testosterone and were not able to extract data on free testosterone from one of these studies, as data were presented in a figure only [36, 37]. One of these RCTs [36] found evidence of a decrease in total testosterone and free testosterone following 12 weeks of vitamin D supplementation. The other RCT [37] showed a 41.2% increase in total testosterone following 8 weeks of vitamin D supplementation.

An MR study by Chen et al., [27] created a genetic risk score, using four single nucleotide polymorphisms (see Table 1) previously found to be associated with vitamin D levels, to estimate its effect on total testosterone in 4254 men. The authors found that using the genetic risk score as an instrument a standard deviation increase in 25-hydroxyvitamin D was associated with an increase in total testosterone levels (Beta-coefficient = 0.12, 95% CI = 0.02–0.22).

### Testosterone-prostate cancer progression studies

Three human cohort studies reported on the association of total testosterone, free testosterone, and dihydrotestosterone on either prostate cancer-specific mortality alone [38] or in combination with the development of metastasis [39] or a Gleason score of ≥ 8 [40]. All three studies were judged at moderate RoB (Table 3). These three cohort studies could not be meta-analysed due to the significant differences in their reported outcomes. We, therefore, describe their results below and in Table 4. No animal studies were identified which examined endogenous testosterone concentrations on measures of prostate cancer progression.

**Table 3:** Risk of bias of testosterone—prostate cancer progression studies

Study	Bias due to confounding	Bias in selection of participants	Bias due to missing data	Bias in measurement of outcome	Bias in measurement of exposure	Bias due to selective reporting	Overall risk
Kjellman 2008	Moderate	Moderate	Low	Low	Low	Moderate	Moderate
Pierorazio 2010	Moderate	Moderate	Low	Low	Low	Moderate	Moderate
Gershman 2014	Moderate	Low	Low	Low	Low	Moderate	Moderate

**Table 4:** Characteristics and results of the included human cohort testosterone-prostate cancer progression association studies

Author (year)	Country	Study name	Number of events/cases	Mean (SD)/median age at baseline (years)	Description of exposures at baseline	Follow-up (years)	Outcome of interest	Results
Kjellman (2008)	Sweden	NA	41/65	65.0 (median)	Dihydrotestosterone measured at diagnosis	12.8 (median)	Prostate cancer-specific mortality	Dihydrotestosterone above 0.67 ng/L (median): reduced mortality (log rank *p* = 0.0075)
Gershman (2014)	USA	Physician’s Health Study and Health Professionals Follow-up Study	106/717	69.1 (7.3)	Pre-diagnostic total testosterone	12.0 (mean)	Lethal prostate cancer (development of metastasis or prostate cancer-specific mortality)	HR1 = 0.95 (95% CI = 0.78 to 1.16, *p* = 0.62)
		Health Professionals Follow-up Study	50/472		Pre-diagnostic free testosterone			HR1 = 0.88 (95% CI = 0.60 to 1.29, *p* = 0.50)
		Physician’s Health Study	85/492		Pre-diagnostic dihydrotestosterone All measured ≥ 2 years before the outcome			HR1 = 1.08 (95% CI = 0.84 to 1.37, *p* = 0.56)
Pierorazio (2010)	USA	Baltimore Longitudinal Study of Aging	36/145	51.6 (15.2)	Repeated pre-diagnostic total testosterone measures	22 years (median)	High-risk disease -prostate cancer with adverse clinical and pathological features including death from disease, a PSA level of > 20 ng/mL or a Gleason sum of ≥ 8	Total testosterone (per ng/dL): HR = 1.002 (95% CI = 0.998 to 1.007, *p* = 0.28)
					Repeated pre-diagnostic free testosterone measures			Calculated free testosterone (per ng/dL): HR = 1.61 (95% CI = 1.18 to 2.204, *p* = 0.003)

Kjellman [38] investigated the effects of dihydrotestosterone measured at the time of a prostate cancer diagnosis on prostate cancer-specific mortality. A sample of 65 men with a median age of 65 years were identified from a population-based prostate cancer screening study with a median follow-up of 12.8 (range 1.1–15.3) years. Men with a biopsy-confirmed diagnosis of prostate cancer were included in Kjellman’s study. The authors reported that men with a median dihydrotestosterone value above 0.67 ng/L had a lower mortality rate than those below the median (log rank *p* = 0.0075).

Gershman [39] examined the effects of pre-diagnostic total and free testosterone and dihydrotestosterone on lethal prostate cancer (defined as development of metastasis or prostate cancer-specific mortality) in men with a mean age of 69 years and a mean follow-up of 12 years. The authors found evidence of an association between total testosterone (HR = 0.95, 95% CI = 0.78–1.16, *p* = 0.62) or free testosterone (HR = 0.88, 95% CI = 0.60–1.29, *p* = 0.50) and a reduced risk of lethal prostate cancer.

Pierorazio [40] evaluated the effects of pre-diagnostic total and free testosterone on high-risk prostate cancer (defined as prostate cancer-specific mortality, a PSA level of > 20 ng/mL or a Gleason score of ≥ 8 at diagnosis) in a cohort study of 145 men with a mean age of 52 years and a median follow-up of 22 years. The authors found evidence of an association between calculated free testosterone (ng/dL) and high-risk prostate cancer (OR = 1.61, 95% CI = 1.18–2.20, *p* = 0.003). There was little evidence of an association between total testosterone (ng/dL) and high-risk prostate cancer (HR = 1.00, 95% CI = 0.998–1.007, *p* = 0.28).

### Grade assessments

We downgraded the certainty of evidence of the effect of vitamin D on total testosterone by two points from high certainty to low certainty due to; the risk of bias in individual studies (two studies were at high risk of bias and five studies had some concerns of bias) (1 point); indirectness of evidence (the studies were from very heterogeneous populations of men including studies in athletes and another in men with chronic heart failure and most were not representative of the target population) (0.5 point); and reporting bias because total testosterone was a primary outcome in only approximately half the number of included studies (0.5 point). We did not downgrade due to imprecision or heterogeneity because there was no evidence of heterogeneity in our meta-analysis and confidence intervals were quite narrow. We downgraded the evidence of the effect of vitamin D on free testosterone by 3 points from high certainty to very low certainty based on the same criteria for the evidence on total testosterone, although with additional points for heterogeneity between the studies included in the meta-analysis (I^2^ = 52.4%) (1 point) and imprecision (1 point). We downgraded the certainty of the evidence of the effect of testosterone on prostate cancer progression from low to very low certainty due to the imprecision of the results (1 point), heterogeneity between studies (1 point) and publication bias (1 point).

## Discussion

We performed a systematic review to investigate whether there was evidence that testosterone concentrations could explain an association of vitamin D with prostate cancer progression. A meta-analysis of 10 RCTs found evidence of a positive association between vitamin D supplementation on total testosterone concentrations. However, we assessed the overall evidence for this association as being of low certainty. We were unable to meta-analyse three studies assessing the association of testosterone with measures of prostate cancer progression. One of the three studies showed an association of pre-diagnostic calculated free testosterone on prostate cancer-specific mortality or advanced prostate cancer (i.e., diagnostic Gleason score of ≥ 8, metastasis). A contradictory finding was observed for dihydrotestosterone which was associated with improved mortality [38]. We assessed the overall certainty of the evidence relating to the association of testosterone with prostate cancer progression as very low.

Our finding of the vitamin D- total testosterone association is supported by a previous published systematic review of 10 RCTs that included 1,061 men [41]. All 10 RCTs were identified in our review, although we excluded one RCT from our review due to strong evidence of a difference in age at baseline between intervention and control groups [42]. We identified 3 additional RCTs [29, 35, 36] which were published since the authors performed their literature searches. In the previous meta-analysis, the authors found little evidence that vitamin D supplementation altered total testosterone levels (mean difference = 0.20, 95% CI = − 0.20–0.60, *p* = 0.336). The 3 additional studies in our review are likely to have increased the precision of our overall result. The authors used weighted mean differences in their meta-analysis, whilst our review using standardised mean differences. However, there is unlikely to be important differences in the magnitude of effect based on these parameters [43]. Another previous review [44] examined this association in men with and without vitamin D deficiency (i.e., 25 (OH)D below 20 ng/mL). Participants included 9892 men with vitamin D deficiency and 10,675 controls from 18 case-control studies. The authors reported a small association between vitamin D and total testosterone (SMD =  − 0.23, 95% CI = − 0.45 to − 0.01; *p* = 0.04). However, all case–control studies were cross-sectional and were at a higher risk of reverse causation, confounding, and measurement error, and the findings should be interpreted with caution.

Due to the small number of studies assessing the association of testosterone with prostate cancer progression, we were unable to draw any strong conclusions. A systematic review by Claps and colleagues [45] investigated the association between total testosterone and overall mortality (including prostate cancer-specific mortality). In their meta-analysis of four cohort studies [38, 39, 46, 47], the authors found little evidence of an association of total testosterone with overall mortality (HR = 1.03, 95% CI = 0.99–1.08, *p* = 0.19). Two of these cohort studies were not included our review as one study reported on men treated with ADT [46] and the other study reported on overall survival [47], not prostate cancer-specific survival. It is, therefore, evident that further research would benefit from examining testosterone concentrations in relation to prostate cancer-specific mortality as well as on measures of advanced prostate cancer (i.e., Gleason scores of ≥ 8, development of metastasises).

### Limitations

There are several limitations with regards to the included studies and their reported outcomes. Almost half of the vitamin D-testosterone studies were judged as having at least some concerns of risk of bias. Few studies reported on the ethnicity of the participants, although all studies were conducted in countries where the population is predominantly white. We can assume that the findings in these studies are not representative of black men who are at increased risk of prostate cancer [48]. There were differences in the definition of prostate cancer progression, and we were unable to meta-analyse studies assessing associations of testosterone with prostate cancer progression due to the heterogeneity in their reported outcomes. All data included in the meta-analysis were from published peer-reviewed articles. We did not contact subject experts regarding any unpublished or published studies which were not identified from our literature searches.

### Implications for future research

We found evidence of an association of increased total testosterone concentrations in men using vitamin D supplementation. However, we found that the overall certainty in the robustness of the finding was low, indicating that further RCTs with total testosterone as a primary outcome with follow-ups of at least 1 year could improve the quality of this evidence. Our review highlights the need for more evidence on the testosterone-prostate cancer progression association. We assessed the certainty of the findings related to this association as very low. Further research could be supported with more cohort studies investigating testosterone as an exposure on well-defined outcomes of prostate cancer progression. Future studies could explore testosterone as a mechanism using large prospective studies which measure vitamin D and testosterone at least 2 years before a diagnosis of prostate cancer. Testosterone could be included in a mediation analysis to assess the effect of vitamin D (exposure) on measures of prostate cancer progression (e.g., PSA or Gleason score as the outcome) through testosterone levels (mediator).

## Conclusion

We found evidence of an effect of vitamin D on circulating total testosterone concentrations in men. We did not find strong evidence of an association of testosterone concentrations on prostate cancer progression. Further research is required to establish whether testosterone is a plausible biological mechanism between vitamin D and prostate cancer progression.

## Supplementary Information

Below is the link to the electronic supplementary material.

Supplementary file1 (PDF 126 kb)

Supplementary file2 (PDF 75 kb)

## Data Availability

The datasets generated during and/or analysed during the current study are available from the corresponding author on reasonable request.

## Code availability

No applicable.
