# Supplementary material for: Does testosterone mediate the relationship between vitamin D and prostate cancer progression? A systematic review and meta-analysis
Source: Cancer Causes Control. 2022 Jun 26;33(8):1025–38. doi: 10.1007/s10552-022-01591-w (PMC9270305; doi:10.1007/s10552-022-01591-w)
Supplement: Supplementary file 2 — Supplementary file2 (PDF 75 kb) [file 10552_2022_1591_MOESM2_ESM.pdf]

Supplementary file 2 – Search strategies from each database

MEDLINE Search strategy:

1. exp Vitamin D/
2. vitamin D?.ti,ab,kf.
3. Vitamin D?.hw.
4. exp Ergocalciferols/
5. ergocalciferols.ti,ab,kf.
6. exp Cholecalciferol/
7. cholecalciferol.ti,ab,kf.
8. exp Calcitriol/
9. calcitriol.ti,ab,kf.
10. Receptors, Calcitriol/
11. vitamin D receptor.ti,ab,kf.
12. (hydroxycholecalciferol? or hydroxy-cholecalciferol? or dihydroxycholecalciferol? or dihydroxy-cholecalciferol? or hydroxyvitamin? D? or hydroxyl-vitamin? D?).ti,ab,kf.
13. 1 or 2 or 3 or 4 or 5 or 6 or 7 or 8 or 9 or 10 or 11 or 12
14. exp Testosterone/
15. testosterone.ti,ab,kf.
16. *Gonadal Hormones/
17. *Gonadal Steroid Hormones/
18. exp Testicular Hormones/
19. exp Testosterone Congeners/
20. 14 or 15 or 16 or 17 or 18 or 19
21. exp Prostatic Neoplasms/
22. (prostat$ adj3 (neoplas$ or cancer$ or carcinoma$ or adenocarcinoma$ or tumo?r$)).ti,ab.
23. (prostat$ and (neoplas$ or cancer$ or carcinoma$ or adenocarcinoma$ or tumo?r$)).kf.
24. 22 or 23
25. exp Neoplasm Metastasis/
26. exp Neoplasm Invasiveness/
27. ((metasta* or (neoplas* or cancer* or carcinoma* or adenocarcinoma* or tumo?r*)) and (invas* or invad* or aggressive* or progressi*)).ti,kf.
28. tumo?r progression.ti,kf.
29. 25 or 26 or 27 or 28
30. exp Prostatic Intraepithelial Neoplasia/
31. 21 or 24 or 30
32. 29 and 31
33. 13 and 20
59. 20 and 32

Pubmed search strategy

Search #1 (vitamin D terms):

"vitamin d"[MeSH Terms] OR "ergocalciferols"[MeSH Terms] OR "vitamin d"[Title/Abstract] OR "ergocalciferols"[MeSH Terms] OR "ergocalciferol*"[Title/Abstract] OR "calcifediol"[MeSH Terms] OR "calcifediol"[Title/Abstract] OR "Calcitriol"[MeSH Terms] OR "Calcitriol"[Title/Abstract] OR "receptors, calcitriol"[MeSH Terms] OR "calcitriol receptor"[Title/Abstract] OR "Cholecalciferol"[MeSH Terms] OR "Cholecalciferol"[Title/Abstract] OR "Hydroxycholecalciferols"[MeSH Terms] OR "Hydroxycholecalciferols"[Title/Abstract] OR "Dihydroxycholecalciferols"[MeSH Terms] OR "Dihydroxycholecalciferols"[Title/Abstract] OR "25 hydroxy vitamin d"[Title/Abstract]

Search #2 (testosterone terms):

"Testosterone"[MeSH Terms] OR "Testosterone"[Title/Abstract] OR "gonadal steroid hormones"[MeSH Terms] OR "gonadal steroid hormone*"[Title/Abstract] OR "gonadal steroid*"[Title/Abstract] OR "gonadal hormones"[MeSH Terms] OR "sex hormone*"[Title/Abstract]

Search #3 (prostate cancer terms):

"prostatic neoplasms"[MeSH Terms] OR "prostatic intraepithelial neoplasia"[MeSH Terms] OR "prostate"[Title/Abstract] OR "prostatic"[Title/Abstract] AND "cancer"[Title/Abstract] OR "carcinoma"[Title/Abstract] OR "adenocarcinoma"[Title/Abstract] OR "tumour"[Title/Abstract] OR "neoplasm"[Title/Abstract] AND "neoplasm metastasis"[MeSH Terms] OR "neoplasm invasiveness"[MeSH Terms] OR "metastas*"[Title/Abstract] OR "invasive*"[Title/Abstract] OR "aggressive*"[Title/Abstract] OR "tumour progressi*"[Title/Abstract]

Search #4 Combine search 1 and 2 with AND

Search #5 Combine search 2 and 3 with AND

BIOSIS Citation Index search strategy

1. ts=vitamin d OR ti=vitamin d
2. ts=ergocalciferol* OR ti=ergocalciferol*
3. ts=calcifediol OR ti= calcifediol
4. ts=calcitriol OR ti= calcitriol
5. ts=cholecalciferol OR ti= cholecalciferol
6. ts=Hydroxycholecalciferol* OR ti= Hydroxycholecalciferol*
7. ts=Dihydroxycholecalciferol* OR ti= Dihydroxycholecalciferol*
8. ts=(hydroxycholecalciferol* OR hydroxy-cholecalciferol* OR dihydroxycholecalciferol* OR dihydroxy-cholecalciferol* OR hydroxyvitamin D OR hydroxyl-vitamin D)
9. ti=(hydroxycholecalciferol* OR hydroxy-cholecalciferol* OR dihydroxycholecalciferol* OR dihydroxy-cholecalciferol*)
10. ts=(25-hydroxy vitamin D OR hydroxyl-vitamin D OR dihydroxyl-vitamin D) OR ti=(25-hydroxy vitamin D OR hydroxyl-vitamin D OR dihydroxyl-vitamin D)
11. ts="Calcitriol receptor"
12. 1 or 2 or 3 or 4 or 5 or 6 or 7 or 8 or 9 or 10 or 11
13. ts=Testosterone OR ti= Testosterone
14. ts="Gonadal Steroid Hormone*" OR ti="Gonadal Steroid Hormone*"
15. ts="Sex Hormone*" OR ti="Sex Hormone*"
16. ts="Gonadal Hormone*" OR ti="Gonadal Hormone*"
17. ts="Gonadal steroid*" OR ti="Gonadal steroid*"
18. 13 or 14 or 15 or 16 or 17
19. ts=(prostat* AND (neoplas* OR cancer OR carcinoma* OR tumor OR adenocarcinoma*)) OR ti=(prostat* AND (neoplas* OR cancer OR carcinoma* OR tumor OR adenocarcinoma*))
20. ts="prostatic intraepithelial neoplasia" OR ti="prostatic intraepithelial neoplasia"
21. ts=(metasta* OR invas* OR invad* OR aggressive* OR progressi*) OR ti=(metasta* invas* or invad* OR aggressive* OR progressi*)
22. #20 OR #19
23. #22 AND #21
24. #18 AND #12
25. #23 AND #18

EMBASE search strategy

1. vitamin d/
2. vitamin D?.ti,ab,kw.
3. Vitamin D?.hw.
4. 24,25 dihydroxyvitamin d/
5. 25 hydroxyvitamin d/
6. *ergocalciferol/
7. ergocalciferol*.ti,ab,kw.
8. exp colecalciferol/
9. colecalciferol.ti,ab,kw.
10. calcitriol/
11. Calcitriol.ti,ab,kw.
12. calcifediol/
13. calcifediol.ti,ab,kw.
14. colecalciferol/
15. colecalciferol.ti,ab,kw.
16. exp vitamin D receptor/
17. exp hydroxycolecalciferol/
18. hydroxycholecalciferol*.ti,ab,kw.
19. hydroxy-cholecalciferol*.ti,ab,kw.
20. exp dihydroxycolecalciferol/
21. Dihydroxycholecalciferol*.ab,kw,ti.
22. 25-hydroxy-vitamin D.ab,kw,ti.
23. exp 25 hydroxyvitamin D/
24. hydroxycholecalciferol.ti,ab,kw.
25. hydroxy-cholecalciferol.ti,ab,kw.
26. exp dihydroxycolecalciferol/
27. dihydroxy-cholecalciferol.ti,ab,kw.
28. or/1-27
29. testosterone/
30. testosterone.ti,ab,kw.
31. *sex hormone/
32. Sex hormone*.ti,ab,kw.
33. sex hormone binding globulin/
34. *androgen/
35. *androgen receptor/
36. or/29-35
37. Prostate cancer/
38. Prostate cancer.ti,ab,kw.
39. Prostate carcinoma/
40. Prostate carcinoma*.ti,ab,kw.
41. Prostate adenocarcinoma/
42. Prostate adenocarcinoma*.ti,ab,kw.
43. Prostate intraepithelial neoplasia/
44. 37 or 38 or 39 or 40 or 41 or 42 or 43
45. exp tumor invasion/
46. metastasis/
47. exp tumor growth/
48. (metasta* or invas* or invad* or aggressive* or progressi*).ti,kw.
49. 45 or 46 or 47 or 48

50. 44 and 49
51. 28 and 36
52. 36 and 50
53. limit 51 to exclude medline journals
54. limit 52 to exclude medline journals
55. limit 51 to embase
56. limit 52 to embase
